# Involvement of Dopamine Receptors in Binge Methamphetamine-Induced Activation of Endoplasmic Reticulum and Mitochondrial Stress Pathways

**DOI:** 10.1371/journal.pone.0028946

**Published:** 2011-12-13

**Authors:** Genevieve Beauvais, Kenisha Atwell, Subramaniam Jayanthi, Bruce Ladenheim, Jean Lud Cadet

**Affiliations:** 1 Molecular Neuropsychiatry Research Branch, National Institute on Drug Abuse, Intramural Research Program, Baltimore, Maryland, United States of America; 2 Faculté de Pharmacie, Université Paris Descartes, Paris, France; National Institutes of Health, United States of America

## Abstract

Single large doses of methamphetamine (METH) cause endoplasmic reticulum (ER) stress and mitochondrial dysfunctions in rodent striata. The dopamine D_1_ receptor appears to be involved in these METH-mediated stresses. The purpose of this study was to investigate if dopamine D_1_ and D_2_ receptors are involved in ER and mitochondrial stresses caused by single-day METH binges in the rat striatum. Male Sprague-Dawley rats received 4 injections of 10 mg/kg of METH alone or in combination with a putative D_1_ or D_2_ receptor antagonist, SCH23390 or raclopride, respectively, given 30 min prior to each METH injection. Rats were euthanized at various timepoints afterwards. Striatal tissues were used in quantitative RT-PCR and western blot analyses. We found that binge METH injections caused increased expression of the pro-survival genes, BiP/GRP-78 and P58^IPK^, in a SCH23390-sensitive manner. METH also caused up-regulation of ER-stress genes, Atf2, Atf3, Atf4, CHOP/Gadd153 and Gadd34. The expression of heat shock proteins (HSPs) was increased after METH injections. SCH23390 completely blocked induction in all analyzed ER stress-related proteins that included ATF3, ATF4, CHOP/Gadd153, HSPs and caspase-12. The dopamine D_2_-like antagonist, raclopride, exerted small to moderate inhibitory influence on some METH-induced changes in ER stress proteins. Importantly, METH caused decreases in the mitochondrial anti-apoptotic protein, Bcl-2, but increases in the pro-apoptotic proteins, Bax, Bad and cytochrome c, in a SCH23390-sensitive fashion. In contrast, raclopride provided only small inhibition of METH-induced changes in mitochondrial proteins. These findings indicate that METH-induced activation of striatal ER and mitochondrial stress pathways might be more related to activation of SCH23390-sensitive receptors.

## Introduction

Methamphetamine (METH) addiction is very prevalent throughout the world. The accumulated evidence suggests that the acute effects of METH on neurons post-synaptic to striatal dopamine (DA) terminals are due to DA release [1] and subsequent stimulation of DA receptors in the brain [2], [3], [4]. Chronic METH abuse is associated with medical, neurologic and neurodegenerative complications [3], [5], [6], [7], [8]. These neuropsychiatric adverse events include secondary depression, psychotic states and psychomotor impairments [9], [10]. Post-mortem studies have revealed that the brains of METH addicts show depletion of DA, serotonin (5-HT) and of their metabolites in the striatum [8]. There are also losses in dopamine transporter (DAT) [7] and serotonin transporter (5-HTT) [11] in the brains of METH abusers. In rodents, METH induces similar degeneration of monoaminergic systems in various regions of the brain including the striatum, cortex and hippocampus [3], [12], [13]. The effects of METH on DA system include reductions in the neurotransmitter, its metabolites, 3,4-dihydroxyphenylacetic acid (DOPAC) and homovanillic acid (HVA), the DA synthesis enzyme, tyrosine hydroxylase (TH), and the vesicular transporter (VMAT2) [3], [4], [13]. The serotonin system is also affected by METH and experiences reduction in the levels of 5-HT, its metabolite, 5-hydroxyindoleacetic acid (5-HIAA), and of the 5-HT synthesis enzyme, tryptophan hydroxylase (TPH) [13]. METH-induced biochemical and structural changes in monoaminergic terminals are dependent on normal dopaminergic functions. Specifically, DA D_1_ and D_2_ receptors antagonists were shown to attenuate the toxic effects of METH on DA and 5-HT systems [4], [13]. In addition, the essential role of DA in METH toxicity was elegantly demonstrated in studies in which depletion of DA provided protection against METH-induced damage of DA terminals whereas increasing DA promoted these toxic effects [14].

METH also causes cell death of neurons located post-synaptic to monoaminergic terminals [2], [3], [4], [12], [15], [16]. Cell death appears to occur in enkephalin-positive cells [16] that express D_2_ receptors [17] and in other neurons [4] that express D_1_ receptors [17]. Although there are multiple classes of DA receptors in the striatum, the most abundant subtypes are the D_1_ and D_2_ receptors [18], [19]. In the dorsal striatum, the D_1_-like subtype of DA receptors is thought to be mainly responsible for METH-induced changes in gene expression and, possibly, for METH-induced neuronal apoptosis [20]. These ideas are consistent with the recent demonstration that activation of endoplasmic reticulum (ER) stress pathways in rat striatum by a single large METH dose is inhibited by the DA D_1_ receptor antagonist, SCH23390 [21]. SCH23390 also blocked METH-induced cell death in the rodent brain [2], [4]. The DA D_2_-like receptor might also be involved in METH-induced cell death because the DA D_2_ receptor antagonist, raclopride, was reported to also inhibit this process to a great degree [4]. Nevertheless, it is still not clear how inhibition of DA D_1_ and D_2_ receptors might interfere with intracellular death pathways in order to protect against METH-induced neuronal apoptosis.

METH is known to exert its toxic effects, in part, by causing oxidative stress [3], [22], [23]. Oxidative stress can increase the expression of ER resident chaperones, such as BiP/GRP-78, P58^IPK^, and heat shock proteins (HSPs) that are important regulators of aberrant protein folding [24]. Under severe ER stress, the ER-located trans-membrane proteins, activating transcription factor 6 (ATF6), inositol-requiring enzyme 1 (Ire-1), and PKR-like ER kinase (PERK) regulate the unfolded protein response (UPR). ATF6 acts as a transcription factor for UPR induction [25]. Phosphorylation of Ire-1 induces ER-resident proteins, such as BIP/GRP-78, GRP94 and C/EBP homologous protein (CHOP)/growth arrest-and DNA damage-inducible gene 153 (Gadd153) [26]. On the other hand, PERK can induce phosphorylation of eukaryotic initiation factor-2α (eIF2α) to inhibit total translation but increases translation of ATF4, which is also a transcription factor for UPR induction [27]. ER stress-induced cellular demise is also mediated, in part, by calpain-mediated activation of the protease caspase-12 [28].

METH administration can also trigger alterations in the expression of the Bcl-2 family of proteins and secondary activation of mitochondria stress-mediated death pathways [29], [30], [31]. Because DA D_1_ and D_2_ antagonists have been shown to provide significant protection against METH-induced cell death [2], [4], we investigated the molecular mechanisms of this protection on METH-induced activation of ER and mitochondrial-dependent pathways in the brain. To pursue this idea further, we used the commonly implemented binge patterns of METH injections employed in toxicity studies for purpose of comparison with previous observations with single METH injections. It was important to do this because single and binge METH injections have been reported to differentially affect striatal neurons [32]. Thus, the first aim of our study was to test the possibility that binge METH administration might cause concomitant changes in ER and mitochondria stresses proteins in the rat striatum. If so, we also wanted to delineate the timing of these METH-induced changes. The second aim was to investigate if the DA D_1_-like receptor antagonist, SCH23390, might block METH-induced changes in expression of genes and proteins involved in both stress pathways. Although SCH23390 can also bind to 5-HT receptors [33], [34], the 5-HT system has not been shown to play an important role in METH toxicity [35], [36]. The third aim was to test if the DA D_2_-like receptor antagonist, raclopride, had any influence on METH-induced alterations in the expression of several proteins involved in ER and mitochondria stress pathways.

## Results

### METH causes early dopamine D_1_ sensitive-up-regulation in mRNA levels of ER stress markers, BiP/GRP-78 and P58^IPK^

**Figure 1:**
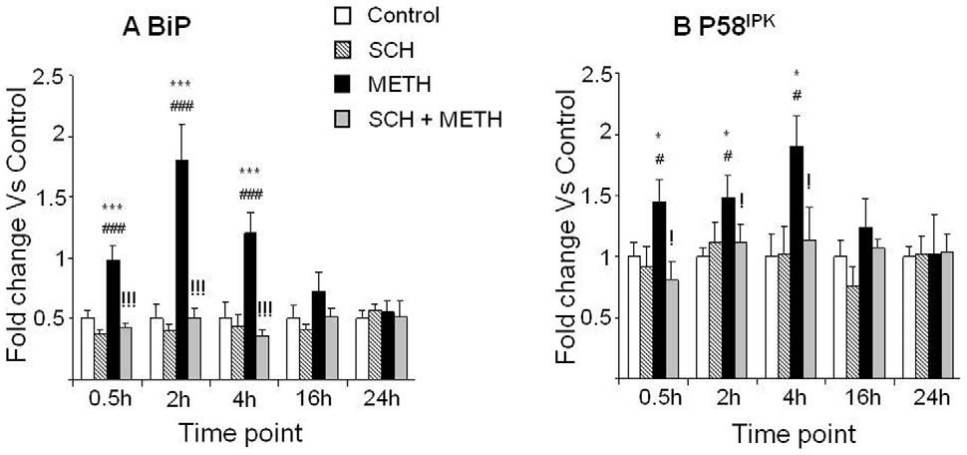
Binge METH injections caused time-dependent increases in the expression of the ER chaperone, BiP/GRP-78, and of the co-chaperone, P58^IPK^. Levels of (A) BiP/GRP-78 and (B) P58^IPK^ transcripts were rapidly increased at 30 min after METH injections. RT-PCR was performed on total RNA isolated from the striatal tissue. Data were obtained from RNA isolated from six animals per group and determined individually. The levels of mRNA were normalized to clathrin mRNA levels. Values obtained for the treatment groups were compared by analysis of variance (ANOVA) followed by post-hoc analyses when ANOVA revealed significant changes. Key to statistics: * = p<0.05; *** = p<0.001, in comparison to the Saline group. # = p<0.05; ### = p<0.001, in comparison to the SCH group. ! = p<0.05; !!! = p<0.001, in comparison of METH group to the SCH+METH group.

Figure 1 shows the effects of METH injections on the expression of the ER stress genes, BiP/Grp-78 [37] and P58^IPK^ [38]. Repeated injections of METH caused very early increases in BiP/Grp-78 mRNA levels which were apparent 30 min after the METH injections and peaked at 2 hr time-point (Fig. 1A). The METH-induced changes were normalized at 16 hr after the last METH injection. METH also caused increases in P58^IPK^ mRNA levels which were also apparent 30 min after the injections, peaked at 4 hr, and then normalized at 16 hr after the binge METH injections (Fig. 1B). SCH23390 prevented the METH-induced increases in BiP/Grp-78 and P58^IPK^ mRNA levels, but had no effects when administered alone (Figs. 1A and 1B).

As a result of these observations, we sought to identify other ER stress proteins whose expression might be influenced by the METH injections. We also examined if these changes might be affected by pretreatment with D_1_-like or D_2_-like receptor antagonists.

### Effects of METH, SCH23390, and raclopride on HSP40 and HSP70 in the rat striatum

**Figure 2:**
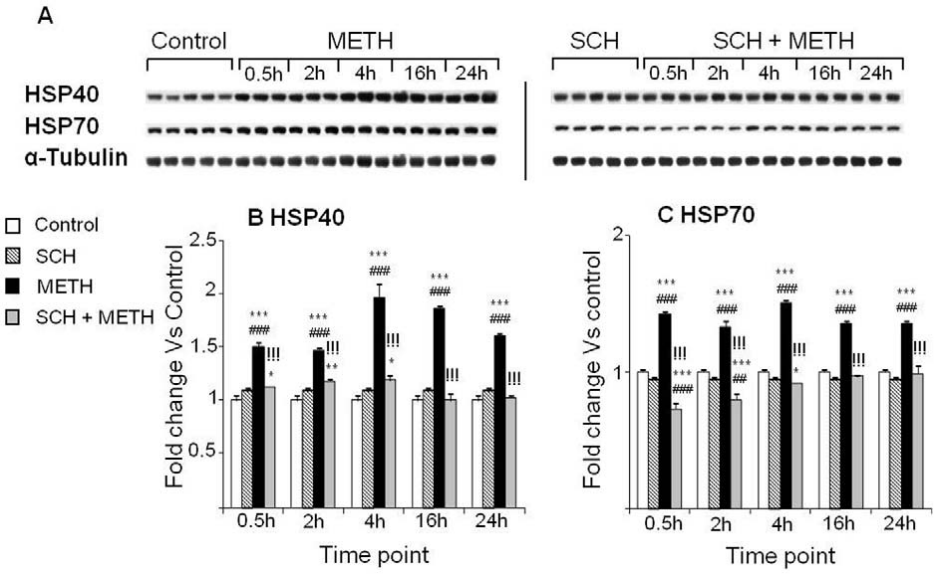
Effects of METH and SCH23390 administration on the expression of cytosolic chaperones HSPs. (A) Representative western blot bands (1 band for Saline or SCH representing each time-point, 3 bands for METH and SCH+METH). METH administration caused rapid and stable induction of the chaperones HSP40 (B) and HSP70 (C). Pretreatment with SCH23390 prevented these increases. Protein expression was normalized to α-Tubulin. Key to statistics: * = p<0.05; ** = p<0.01; *** = p<0.001, in comparison to the Saline group. ## = p<0.01; ### = p<0.001, in comparison to the SCH group. !!! = p<0.001, in comparison of METH group to the SCH+METH group.

We used western blot experiments in order to determine if METH or the D_1_ receptor antagonist, SCH23390, had any effects on the expression of the chaperones, HSP40 and HSP70. Figure 2 shows that binge METH injections caused significant and prolonged increases in the expression of HSP40 (Figs. 2A and 2B) and HSP70 (Figs. 2A and 2C) protein levels in the rat striatum. SCH23390 pretreatment completely blocked METH-induced increases in HSP40 (Fig. 2B) and HSP70 (Fig. 2C) at all time points.

**Figure 3:**
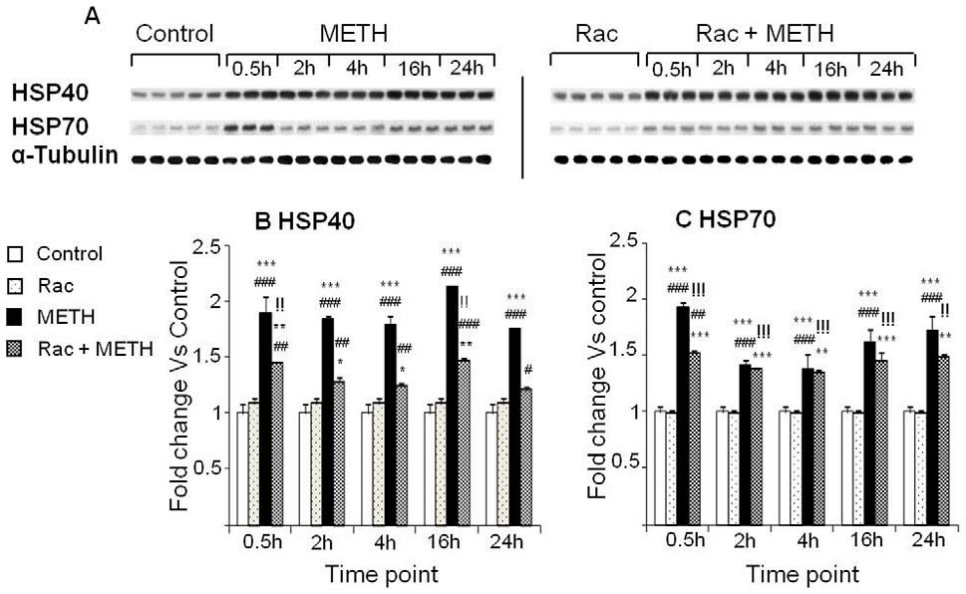
Effects of raclopride on METH-induced HSP40 and HSP70. (A) Representative immunoblots of the effects of the drugs. (B, C) Quantitative analysis of the proteins. Protein expression was normalized to α-Tubulin. Key to statistics: * = p<0.05; ** = p<0.01; *** = p<0.001, in comparison to the Saline group. # = p<0.05; ## = p<0.01; ### = p<0.001, in comparison to the Rac group. !! = p<0.01; !!! = p<0.001, in comparison of METH group to the Rac+METH group.

The effects of the D_2_-like receptor antagonist, raclopride, on METH-induced HSPs proteins were also investigated in a different group of rats. METH alone caused rapid and persistent increases in HSP40 and HSP70 (Fig. 3). Raclopride pretreatment caused significant attenuation of METH-induced changes in HSP40 expression at all time-points examined in the present study (Fig. 3B). However, injections of raclopride in combination with METH only slightly attenuated the METH-induced increases in HSP70 at 30 min, but were ineffective afterwards (Fig. 3C).

### Effects of METH and SCH23390 on mRNA levels of ER stress responsive genes

**Figure 4:**
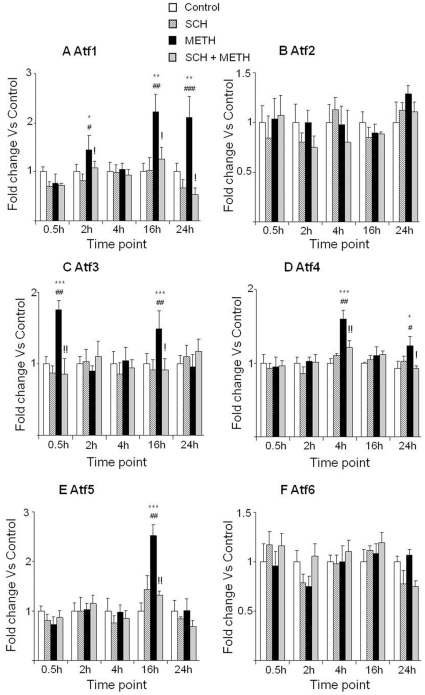
METH caused differential effects on ER stress genes. Binge toxic doses of METH have differential effects on the members of the ATF family of transcription factors (A–F). Key to statistics: * = p<0.05; ** = p<0.01; *** = p<0.001, in comparison to the Saline group. # = p<0.05; ## = p<0.01; ### = p<0.001, in comparison to the SCH group. ! = p<0.05; !! = p<0.01, in comparison of METH group to the SCH+METH group.

We also measured the effects of METH on the mRNA levels of several members of the activating transcription factor (ATF) family (Fig. 4). Injections of METH caused delayed increases in Atf1 (16 and 24 hr) (Fig. 4A) and Atf5 (16 hr) (Fig. 4E) mRNA levels. There were bimodal increases in Atf3 (Fig. 4C) (0.5 and 16 hr) and Atf4 (Fig. 4D) transcripts that occurred at 4 and 16 hr after METH. SCH23390 pretreatment blocked these METH-induced increases (Figs. 4A and 4C–4E). The mRNA levels of Atf2 and Atf6 (Figs. 4B and 4F) were not affected by any of the drug combinations.

**Figure 5:**
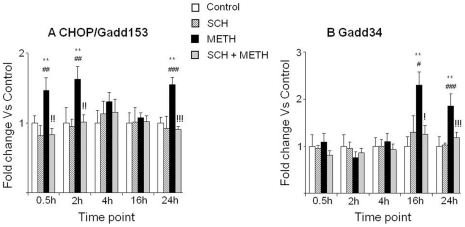
Effects of METH on the transcript levels of pro-death genes. (A) METH caused rapid induction in Chop/Gadd153 mRNA levels. (B) Gadd34 was up-regulated at late time-points. Key to statistics: ** = p<0.01, in comparison to the Saline group. # = p<0.05; ## = p<0.01; ### = p<0.001, in comparison to the SCH group. ! = p<0.05; !! = p<0.01; !!! = p<0.001, in comparison of METH group to the SCH+METH group.

METH injections also caused biphasic changes in the mRNA levels of CHOP, consisting of rapid increases 30 min after METH injection, peaking at 2 hr, and returning to normal by 4 hr after METH administration. Unexpectedly, there were also delayed METH-induced increases in CHOP mRNA levels at 24 hr after the last drug injection (Fig. 5A). As shown in Figure 5A, SCH23390 pretreatment blocked METH-induced changes in CHOP mRNA levels. METH injections also caused increases in Gadd34 mRNA expression that were apparent at 16 and 24 hr, in a SCH23390-sensitive manner (Fig. 5B).

### Effects of METH, SCH23390, and raclopride on ER stress-related proteins in the rat striatum

**Figure 6:**
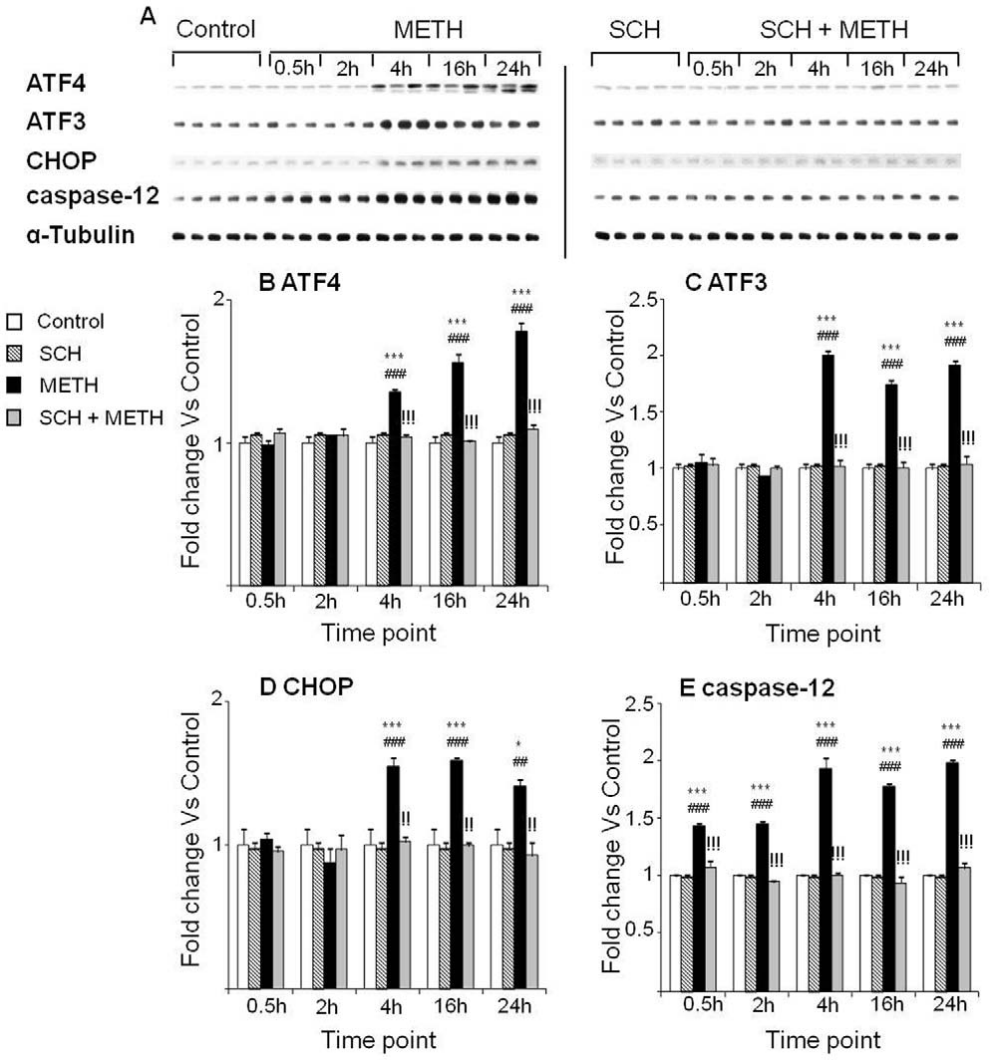
Effects of METH injections and SCH23390 treatment on the expression of stress response regulated proteins. (A) Representative immunoblots showing the effects of METH and SCH23390. (A–E) Pretreatment with SCH23390 blocked the METH-induced changes on ATF3 (B), ATF4 (C), CHOP (D) and caspase-12 (E). Protein expression was normalized to α-Tubulin. Key to statistics: * = p<0.05; *** = p<0.001, in comparison to the Saline group. ## = p<0.01; ### = p<0.001, in comparison to the SCH group. !! = p<0.01; !!! = p<0.001, in comparison of METH group to the SCH+METH group.

Figure 6 shows the effects of METH on ATF3 and ATF4 protein levels which were determined by western blot. Binge METH injections caused somewhat delayed increases in ATF3 (Figs. 6A and 6B) and ATF4 protein levels at 4, 16, and 24 hr (Figs. 6A and 6C). Pretreatment with SCH23390 prevented METH-induced changes in both ATF3 and ATF4 protein levels (Figs. 6A–6C). Figures 6A and 6D show that there were also METH-induced increases in CHOP protein levels which remained elevated from 4 hr after the last METH injection and thereafter. As shown in Figure 6D, SCH23390 pretreatment blocked METH-induced changes in CHOP protein levels. Figure 6E illustrates the effects of binge METH injections on cleaved caspase-12 protein expression. There were METH-induced increases in cleaved caspase-12 at all time points after the last injection of the drug (Figs. 6A and 6E). SCH23390 pretreatment also prevented the METH-induced changes in caspase-12 protein.

**Figure 7:**
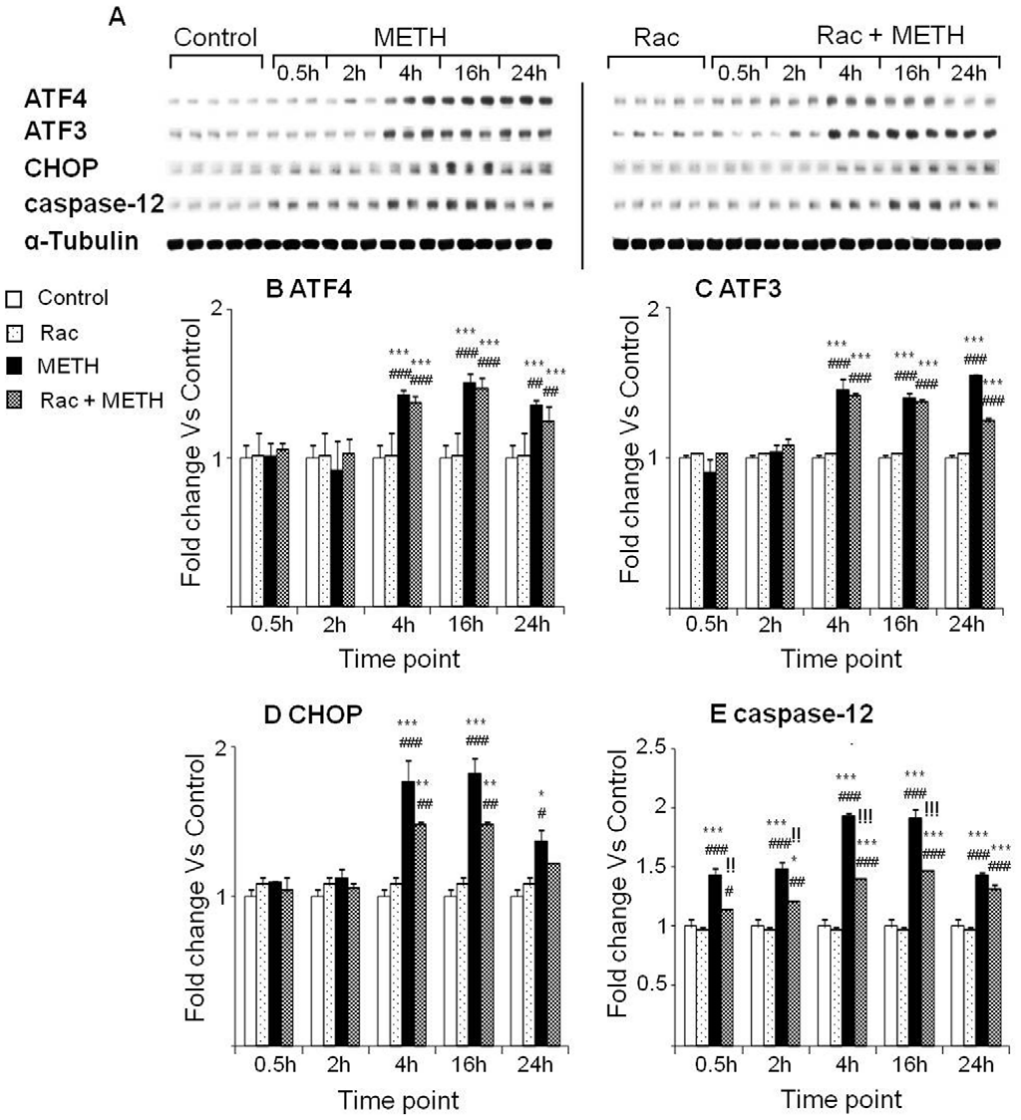
Raclopride did not block METH-induced ATF4 and ATF3 expression. (A) Representative immunoblots of ATF4, ATF3, CHOP and caspase-12. Quantification of ATF4 (B), ATF3 (C), CHOP (D) and caspase-12 (E) are shown. Key to statistics: * = p<0.05; ** = p<0.01; *** = p<0.001, in comparison to the Saline group. # = p<0.05; ## = p<0.01; ### = p<0.001, in comparison to the Rac group. !! = p<0.01; !!! = p<0.001, in comparison of METH group to the Rac+METH group.

In the experiments testing the effects of raclopride, we confirmed the METH-induced increases in ATF3, ATF4, CHOP and caspase-12 protein expression (Fig. 7). Pretreatment with raclopride had no significant effects on the METH-induced increases in ATF3 and ATF4 protein levels (Figs. 7B and 7C). Raclopride pretreatment caused partial attenuation of the METH-induced effects on CHOP and caspase-12 expression (Figs. 7D and 7E).

### Effects of METH, SCH23390, and raclopride on proteins involved in mitochondria-dependent stress pathways

**Figure 8:**
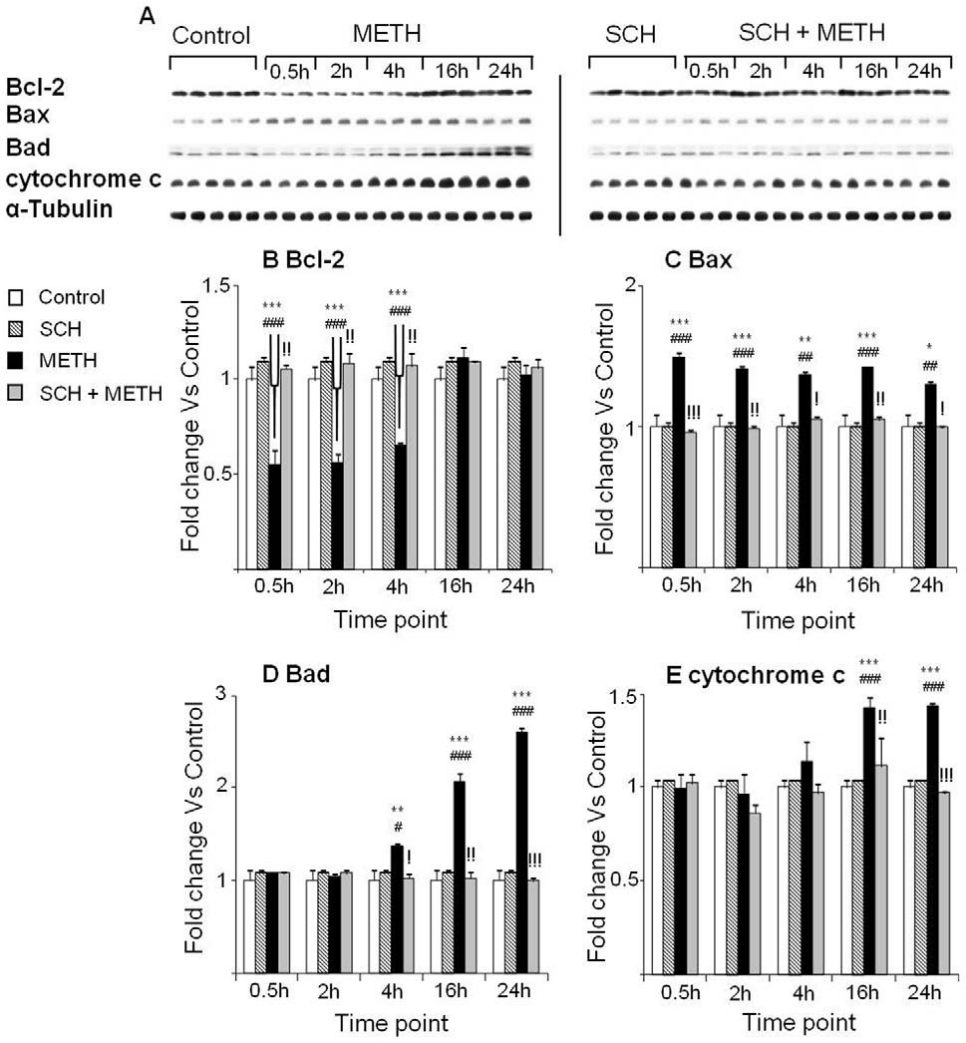
Effects of METH and SCH23390 treatments on the expression of mitochondrial dysfunction-related proteins. (A) Representation of immunoblots of Bcl-2, Bax, Bad and cytochrome c. (B) METH injections caused rapid SCH23390-sensitive decreases in Bcl-2 protein levels. METH caused rapid increases in (C) Bax and (D) Bad protein levels that were inhibited by SCH23390 pretreatment. (E) METH injections were associated with release of cytochrome c from mitochondrial to cytoplasmic compartments, as shown by increases in cytochrome c levels in cytoplasmic fractions. Pretreatment with SCH23390 blocked cytochrome c release. Key to statistics: * = p<0.05; ** = p<0.01; *** = p<0.001, in comparison to the Saline group. # = p<0.05; ## = p<0.01; ### = p<0.001, in comparison to the SCH group. ! = p<0.05; !! = p<0.01; !!! = p<0.001, in comparison of METH group to the SCH+METH group.

Binge METH injections caused significant decreases in Bcl-2 protein levels at 30 min, 2 hr and 4 hr in rats treated with METH alone (Figs. 8A and 8B). METH also caused significant increases in Bax protein levels (Fig. 8C). The drug also caused more delayed increases in Bad protein expression which were apparent at 4 hr (Fig. 8D). Rats pretreated with the D_1_ antagonist, SCH23390, showed significant attenuation of the METH-induced changes in Bcl-2, Bax and Bad protein expression (Figs. 8A–8D). We also measured the effects of METH on cytochrome c because this protein is involved in the mitochondria-dependent death pathway and is released from mitochondria into the cytoplasmic during the process of apoptosis caused by various agents [39], [40]. METH caused increases in cytosolic cytochrome c protein levels at 16 hr and 24 hr time-points that were inhibited by pretreatment with SCH23390 (Fig. 8E).

**Figure 9:**
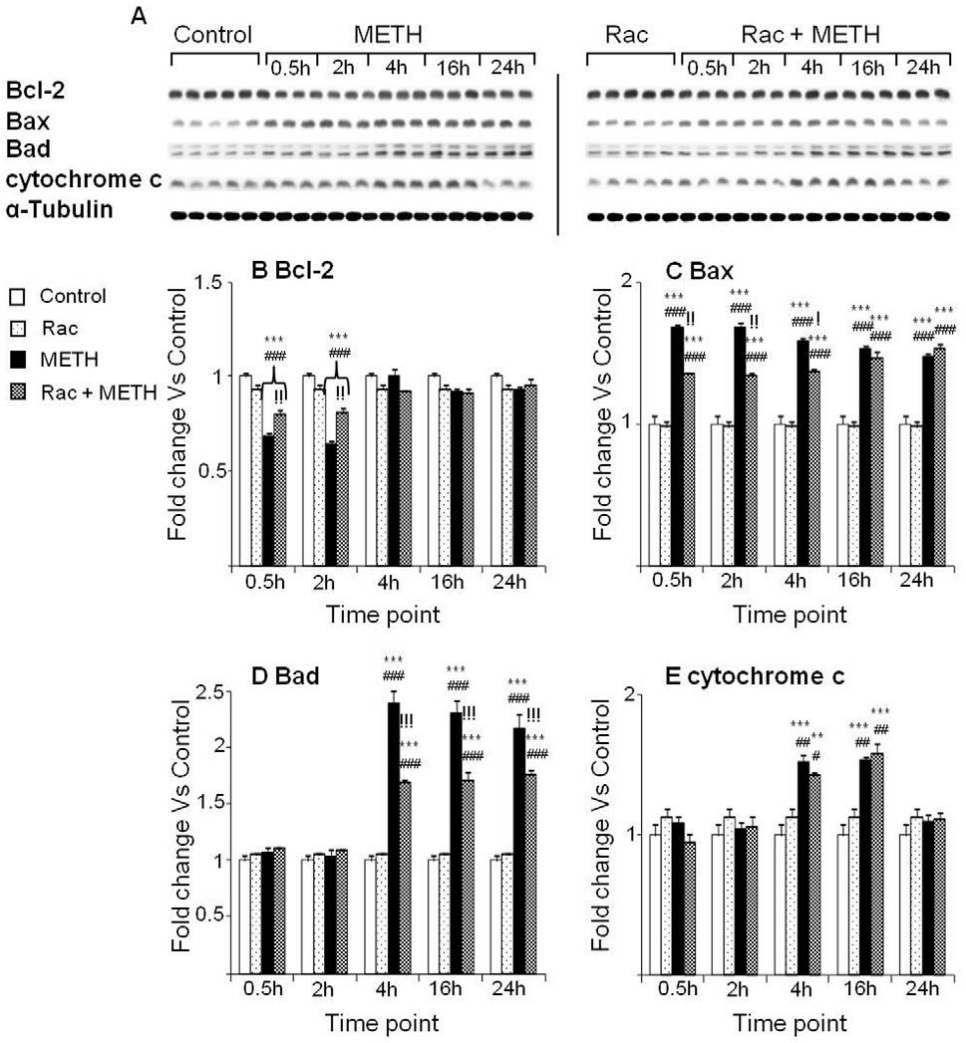
The effects of METH and raclopride on the expression of the Bcl-2 family of proteins and cytochrome c. (A) Representative immunoblots. (B) Pretreatment with raclopride attenuated METH-induced decreases in Bcl-2 protein levels, and (C, D) increases in Bax and Bad expression. In contrast, raclopride was ineffective to block cytochrome c induction (E). Protein expression was normalized to α-Tubulin. Key to statistics: ** = p<0.01; *** = p<0.001, in comparison to the Saline group. # = p<0.05; ## = p<0.01; ### = p<0.001, in comparison to the Rac group. ! = p<0.05; !! = p<0.01; !!! = p<0.001, in comparison of METH group to the Rac+METH group.

We also tested the effects of METH and raclopride on proteins that are involved in mitochondria-dependent cellular stress. Rats injected with METH showed significant decreases in Bcl-2 protein levels at both 30 min and 2 hr after the last injection of METH (Fig. 9B). METH caused increases in Bax protein levels that lasted throughout the experiments (Fig. 9C). Bad protein levels were also increased (Fig. 9D). The expression of cytochrome c was also increased in the cytosol at 4 hr and 16 hr after METH injections (Fig. 9E). Raclopride caused only small attenuation of the effects of METH on Bcl-2 expression (Fig. 9A). In addition, the D_2_-like receptor antagonist attenuated the METH-induced early effects but not its later effects on Bax expression (Fig. 9B). Raclopride had some preventive effects on METH-induced Bad protein (Fig. 9D) but failed to impact METH-induced increases in cytosolic cytochrome c protein levels (Fig. 9E).

### Effects of SCH23390 and raclopride pretreatment on METH-induced hyperthermia

**Figure 10:**
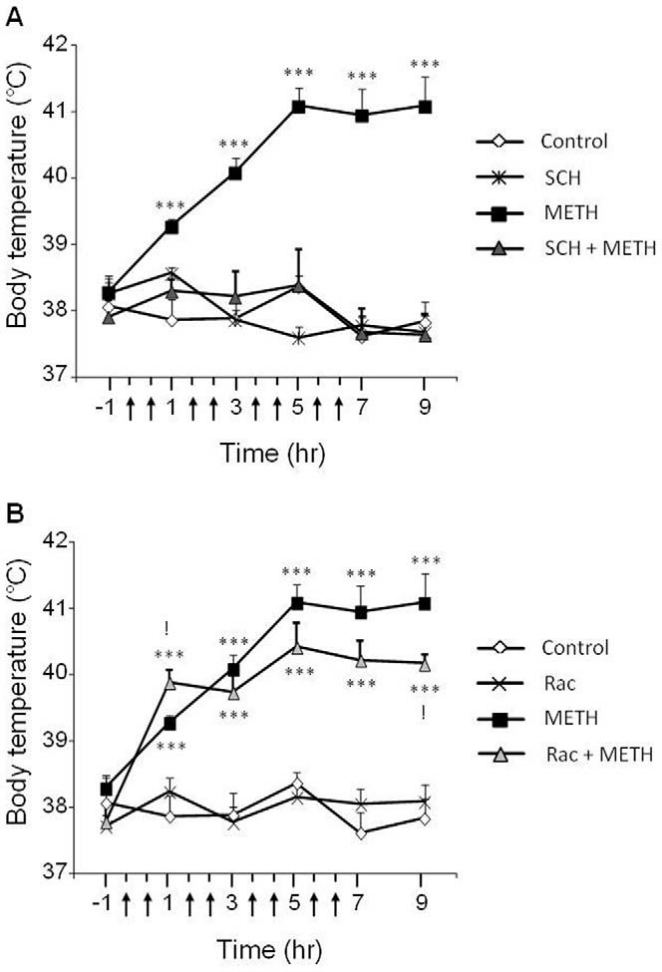
Effects of SCH23390 and raclopride on METH-induced hyperthermia. Animals in the control group received an injection of saline followed 30 min later by another injection of saline, this pattern of injections was repeated four times at 2-hr intervals. The METH treatment group of rats received four injections of saline at 2-hr intervals, each saline injection being followed by an injection of METH (10 mg/kg). (A) Two other groups of animals were pretreated with SCH23390 30 min before each of four saline or METH injections given according to the intervals described above. (B) Two other groups were pretreated with raclopride and treated with either saline or METH injections as above. Temperature was recorded 1 hr prior to the first injection (−1 hr), 30 min after each combined set of injections (shown in arrows), and 2 hr after the final injection. Statistical differences in temperature were considered significant at p values less than 0.05. Key to statistics: *** = p<0.001, in comparison to the control group. ! = p<0.05, in comparison to the METH group (post-hoc test).

In order to test for possible contributions of hyperthermia to our results, we also measured temperature in all experimental groups. As expected, binge METH injections caused significant increases in rat body temperature (Fig. 10). Hyperthermia was apparent 30 min after the first injection of METH (10 mg/kg), stayed elevated, and was still present 2 hr after the last injection (Fig. 10A). The average temperature of rats increased from 37.9°C to 39.3°C (p<0.0001) with the highest temperature reaching 41.08°C (p<0.0001) after the third METH injection. Pretreatment with SCH23390 completely blocked METH -induced hyperthermia (Fig. 10A).

Figure 10B shows the effects of raclopride on METH -induced hyperthermia in rats. Post-hoc analysis revealed that raclopride combined with METH caused a higher increase in temperature than METH alone (+1°C, p = 0.0243) after the first injection of METH (Fig. 10B). That group showed slightly lower but not significant decreases in temperature in comparison to the METH alone group, except for the last time point. At all time points, the METH plus raclopride group showed much higher levels of body temperature than the control group (Fig. 10B).

## Discussion

METH-induced excessive release of DA results in the formation of reactive oxygen species that damage terminals of DA neurons [22], [41]. METH also causes neuronal apoptosis in neurons post-synaptic to DA terminals [2], [12]. These deleterious effects appear to be mediated, in part, by oxidative stress as well as by mitochondrial and ER stresses [3], [42], [43], that are secondary to increased DA overflow in the synaptic cleft [1]. In addition to the processes described before, METH-induced toxicity and molecular events, appear to also depend mainly on stimulation of DA D_1_-like receptors [20], with DA D_2_-like receptors also playing a role in preventing METH-induced neuronal apoptosis [4]. It was, therefore, important to attempt to dissect the role of these subtypes of receptors on signaling mechanisms that have been shown to participate in METH-induced demise of striatal neurons located post-synaptic to DA nerve endings [3].

In the present report, we used the relatively selective DA D_1_ receptor antagonist, SCH23390 [44] and the somewhat, more selective DA D_2_ receptor antagonist, raclopride [45]. In addition to its antagonistic properties on the D_1_-like receptor, SCH23390 has high affinity for other receptors. For example, SCH23390 binds to the DA D_5_ receptor [46], but this receptor has lower level of expression than the D_1_ receptor in the striatum [47]. SCH23390 has high affinity (IC_50_ = 30 nM) for 5-HT_2_ receptors that are abundant in the striatum, being 4.5–23 times less potent than the reference 5-HT_2_ receptor antagonists [33]. Injections of increasing doses of SCH23390 (0.03–10.0 mg/kg intra-peritoneally) blocked binding of the serotonergic receptor antagonist, (^3^H)-spiperone, to 5-HT_2_ receptors in vivo in the frontal cortex but not in the striatum of rats [34]. Importantly, single (0.1 mg/kg or 5 mg/kg) or repeated administration of SCH23390 (0.1 mg/kg daily for 21 days) did not alter the kinetic characteristics of 5-HT_2_ receptors, 5-HT levels, or 5-HT turnover [35]. Since we used a total of 2 mg/kg of SCH23390 per animal in the present experiment, it is possible that the drug might be affecting mainly striatal DA D_1_ receptors. Most importantly, METH-induced biochemical and structural abnormalities in the striatum do not appear to depend on 5-HT neurotransmission because 5-HT_2_ and 5-HT_3/4_ receptor antagonists did not prevent METH-induced reductions in markers of monoaminergic neurons and of TH or TPH activities [48]. Moreover, increasing 5-HT levels by using the 5-HT precursor, 5-hydroxytryptophan, or decreasing 5-HT with the reversible inhibitor of TPH, p-chlorophenylalanine, did not influence METH-induced reductions of DA, TH or DAT levels [36]. Furthermore, deletion of the TPH2 gene that caused marked 5-HT depletion in mice also did not impact the toxic effects of METH on striatal dopaminergic markers [36]. Thus, when taken together with these observations, our present results suggest that SCH23390 might be exerting its protective effects against ER and mitochondria stresses mainly by inhibiting striatal DA D_1_-like but not 5-HT_2_ receptors. Further studies using knockout mice might help to clarify these issues further. Similar to our use of SCH23390, we used raclopride in our experiments based on its specificity to antagonize DA D_2_ receptors. However, raclopride has higher affinity at D_2_ than at D_3_ receptors [45], [49], [50]. Although D_2_ and D_3_ receptors are members of the D_2_-like family of receptors, they have differential anatomical distribution in the brain. D_2_ receptors are abundant in the dorsal part of the striatum, containing the caudate and putamen; while D_3_ receptors are more concentrated in limbic areas like the nucleus accumbens, the ventral part of the striatal nucleus [51], [52]. Because the present deals with the dorsal striatum, it is likely that our results are due to D_2_ receptor antagonism.

The ER is a highly versatile protein synthesis factory that maintains cellular homeostasis via tight regulation of constitutive and inducible ER resident chaperones [53], [54]. BiP/GRP-78, a glucose-regulated and calcium binding ER chaperone protein, is a central regulator of the UPR [37]. Increased availability of BiP/GRP-78 in the lumen of the ER helps or influences translocation of new synthesized proteins [55]. In the present study, we observed early increases in BiP/Grp-78 mRNA levels, in a fashion consistent with similar changes reported after a single injection of a large dose (40 mg/kg) of METH [21], [56], after METH self-administration [57], and after multiple injections of amphetamine (AMPH) [58]. We also identified similar SCH23390-sensitive increases in the mRNA levels of the ER membrane chaperone, P58^IPK^. Because up-regulation of P58^IPK^ mRNA is a common response to ER stress events [38], [59], the present findings suggest that binge METH injections might cause ER stress. P58^IPK^ is thought to be a co-chaperone which interacts with BiP/GRP-78 and other cytosolic chaperones including HSPs to promote co-translocational ER protein degradation [59], [60], [61]. P58^IPK^ can also independently bind to and inhibit the ER stress-inducible eIF2α kinase, PERK, in order to attenuate the UPR cascade via negative feedback [38]. Thus, our observations of METH-induced simultaneous increases in BiP/Grp-78 and P58^IPK^ suggest that the organism was triggering compensatory responses to fight against METH-induced ER stress events.

We found, in addition, that binge METH injections caused significant increases in HSP40 and HSP70, chaperones that function to assist in the folding of stress-denatured proteins and have anti-apoptotic properties [62]. Our observations of METH-induced early increases in the levels of these two proteins are analogous to those reported in previous studies which showed that single toxic METH injections can cause increases in HSP70-like proteins [42], [63], [64]. Moreover, binge patterns of AMPH injections were also found to cause significant increases in the expression of both HSP40 and HSP70 proteins in the vasculature surrounding the forebrain [58]. Thus, our observations extend these findings by showing that both relatively selective D_1_ and D_2_ receptor antagonists can attenuate METH-induced expression of HSPs, suggesting the involvement of both subtypes of DA receptors in mediating these increases. Previous studies have shown that the HSP induction after METH administration depends on METH-induced hyperthermia [42], [65], [66]. These results are consistent with our present observations that the METH injections cause both hyperthermia and HSP induction. METH-induced changes in HSPs can be blocked by preventing hyperthermia in mice treated with ibogaine [66]. In addition, lowering ambient temperature to 18°C attenuated the hyperthermic response to METH and blocked HSP72 induction [65]. Our results thus suggest that the blocking effects of SCH23390 for METH-induced HSP40 and HSP70 chaperones might be dependent, in part, on the prevention of METH-induced hyperthermia by SCH23390, since pretreatment with raclopride, which did not prevent METH-induced increases in body temperature, still provided some degree of inhibition of HSP induction by METH. The blocking effects of raclopride might be due, in part, to its inhibitory effects on the ER stress pathway because activation of that pathway can also result in increased HSP mRNA levels [67]. Taken together, our present observations suggest that multiple factors might be involved in HSP regulation. This suggestion might explain the biphasic induction of HSP70 observed after METH since HSP70 mRNA levels were elevated at 30 min post-METH treatment, became normalized in the intervening hours, and then increased again at 16 and 24 hr after the last METH injection.

We found that binge METH injections caused biphasic pattern of induction of Atf1, Atf3 and Atf4 genes. Members of the activating transcription factor (ATF) family have been implicated in various stress responses [68], [69]. Members of the ATF/CREB family are immediate early responsive genes that are regulated through a cAMP responsive element (CRE) consensus binding site [68]. The transcription factors in the ATF and activating protein-1 (AP-1) families can dimerize through their basic leucine-zipper domain and regulate their own expression [70]. Thus, it is possible that the early and transient induction of transcription of Atf1 and Atf3, after METH administration, might be due to their regulation by CREB and AP-1 proteins. ATF1 has a high degree of homology with CREB with which it can heterodimerize. However, the role of ATF1 transcription factor in response to ER stress has not been investigated [71]. In contrast, there are several reports of the role of ATF3 and ATF4 in the ER stress response pathway [67], [69]. Our results showed a delayed induction of Atf3 gene, an effect that might be regulated by ATF4, as previously reported in other ER stress models [69]. There was also a rapid induction of Atf4 mRNA at 4 h, followed by some degree of normalization, and then a delayed induction at 24 h after the last METH injection. The changes in Atf3 and Atf4 mRNA levels were also associated with increases in both ATF3 and ATF4 protein levels, findings consistent with our observations following a single large dose of METH [21]. It is also important to point out that the induction of these members of the ATF/CREB family might be regulated by CREB that is downstream of the D_1_-cAMP-PKA cascade [72]. This idea is consistent with our findings that putative blockade of the DA D_1_ receptor by SCH23390 can completely block METH-induced ATF3 and ATF4 protein expression. ATF4 also regulates the expression of CHOP/Gadd153 during the UPR [73]. The CHOP promoter contains C/EBP-ATF and ER stress responsive element (ERSE) sites that are essential for CHOP induction during ER stress [74], [75]. During ER stress, ATF4 binds to C/EBP-ATF on the CHOP promoter, providing a partial explanation of the biphasic nature of METH-induced CHOP induction [73], [75]. This discussion suggests that multiple mechanisms might contribute to METH-induced ER stress, since we also observed changes in SCH23390-sensitive cleaved caspase-12 proteins after METH administration, with only partial inhibitory effects observed after pre-treatment with raclopride. The potential role of temperature regulation in these METH-induced changes needs to also be taken into consideration since SCH23390, but not raclopride, was able to block METH-induced hyperthermia.

Mitochondrial dysfunctions have been reported to influence METH toxicity [56], [76]. METH-induced cell death involves the release of the apoptogenic molecules cytochrome c and apoptosis inducing factor (AIF) from mitochondria, upregulation of pro-death members of the Bcl-2 family of mitochondrial proteins, as well as downregulation of anti-death proteins [31], [56]. Over-expression of Bcl-2, an anti-apoptotic gene, was able to protect against METH-induced apoptosis in immortalized neural cells [29]. The present findings of METH-induced decreases in the anti-apoptotic protein, Bcl-2, but increases in the pro-apoptotic proteins, Bax and Bad, are consistent with those observed after single large doses of METH [31]. Our results also suggest that these changes seem to be dependent on SCH23390-sensitive receptors but not on raclopride-sensitive ones.

Taken together, our findings suggest that SCH23390 and raclopride provide differential inhibition on METH-induced changes in proteins involved in ER and mitochondria cell death pathways. Our observations might provide a partial explanation for the previous report that SCH23390 provided almost total protection against cell death at a relatively low dose (0.1 mg/kg) given 30 min before an injection of METH (30 mg/kg) [4]. However, comparatively higher dose of raclopride (1 mg/kg) was required to observe similar protective effects [4]. Therefore, it is possible that the dosage of raclopride used in the present study might not have been enough to block METH-induced changes in ER stress-related genes and mitochondrial proteins, so that higher raclopride doses might have been more effective. However, use of such higher doses might cause a loss of the D_2_/D_3_ specificity of the drug. The possibility also exists that differences in paradigms used in the two different studies, [single injection in the previous study [4] and multiple injections in our study], might have caused some of the discrepancies in the observations. It also needs to be pointed out that measurements of TUNEL-positive cells [4] are not equivalent to measures of ER- and mitochondria-dependent pathways (present study). Blockade of ER-dependent pathways by higher doses of raclopride might be sufficient to block the appearance of TUNEL-positive cells [4]. This remains to be determined.

In summary, we report, for the first time, that binge METH injections can cause substantial increases in the expression of proteins that participate in ER- and mitochondria-dependent stress responses. These METH-induced changes appear to be secondary, for the most part, to stimulation of receptors that are more sensitive to inhibition by SCH23390 that almost completely blocked the METH-induced alterations in proteins involved in both ER and mitochondrial stresses. In contrast, the D_2_-like receptor antagonist, raclopride, had small to moderate effects on ER stress proteins but had no significant effects on mitochondria-dependent cellular stress proteins. When taken together, our results and those of other investigators suggest that the protective effects of SCH23390 might be due to inhibition of multiple death pathways in various subtypes of striatal neurons [4], [77] whereas raclopride might attenuate METH-induced activation of ER stress in enkephalin-positive GABA neurons [4], [16] that express mainly the DA D_2_ receptor subtype [17].

## Materials and Methods

### Animals, Drug Treatment and tissue collection

The drugs used are (+/−)-methamphetamine HCL (NIDA Pharmacy), SCH23390 hydrochloride (TOCRIS bioscience, Ellisville, MO, USA) and raclopride (Sigma Aldrich, St. Louis, MO, USA). All drugs were diluted with 0.9% saline. All experiments were according to the NIH Guide for the Care and Use of Laboratory Animals and were approved by the local Animal Care Committee.

Male Sprague-Dawley rats (Charles River Labs, Raleigh, NC, USA), weighing 250–300 g, were housed individually in cages in a temperature-controlled room (22°C) and had free access to food and water. To test the effects of the D_1_ receptor antagonist, SCH23390, animals were divided in four treatment groups. One group received four intra-peritoneal injections of saline given at 2-hr intervals and followed each by a dose of 10 mg/kg of METH 30 min later. Another group received saline alone according to the same schedule. The third and fourth groups received injections of SCH23390 (0.5 mg/kg) 30 min before each injection of saline or METH. The dose of SCH23390 used, was based on its high affinity for D_1_ receptors (K_i_ = 0.14 nM) [44]. Thus, the four groups were: Saline+Saline (Control), SCH23390+Saline (SCH), Saline+METH (METH) and SCH23390+METH (SCH+METH), administered as patterns repeated four times at intervals two hours. Tympanic temperatures of the rats were measured with a Vet-Temp Instant Animal Ear Thermometer. Temperature was recorded half-hour after each pattern of injections, and two hours after the last injection. Rats were sacrificed by decapitation at 30 min, 2, 4, 16 and 24 hr after the last saline or METH injections. Their brains were rapidly removed; striatal tissues were dissected, placed on dry ice, and then stored at −80°C until further assays. One side of the brain was used for quantitative PCR and the other side for western blot analyses.

Studies on the effects of D_2_ receptor antagonism were conducted in a second group of rats. Experiments were, for the most part, similar to the ones described for SCH23390, except for the fact that we used the D_2_-like receptor antagonist, raclopride (K_D_ = 1 nM) at a dose of 0.5 mg/kg administered four times [45], [49]. We also focused mostly on protein expression since protein products are the responsible agents in biochemical pathways. There were four groups of animals: Saline+Saline (Control), raclopride+Saline (Rac), Saline+METH (METH) and raclopride+METH (Rac+METH). Tympanic temperatures of the animals were also measured at the times mentioned earlier.

### Quantitative RT-PCR analysis

Total RNA was extracted from striatal samples and used for quantitative PCR to measure the expression of ER stress genes. We used the Qiagen RNeasy Midi kit (Qiagen, Valencia, CA, USA) to isolate total RNA. Analysis of samples for quality and quantity was assessed using an Agilent 2100 Bioanalyzer (Agilent, Palo Alto, CA, USA). A total of 1 µg RNA per sample was reverse-transcribed using oligo (dT) into cDNA using Advantage RT for PCR kit (Clontech, Mountain View, CA, USA). Sequences for gene-specific primers were designed by the LightCycler probe design software v. 2.0 (Roche, Indianapolis, IN, USA) and purchased from Synthesis and Sequencing Facility of Johns Hopkins University (Baltimore, MD USA). These sequences are listed in the Table 1. PCR experiments were performed using Lightcycler 480 II (Roche, Indianapolis, IN, USA) and iQ SYBR Green Supermix (Roche, USA). We have used a total of six animals per group in our experiment and have replicated each PCR running two or three times. Quantitation of our samples was determined using the second derivative crossing-points analysis. We have used the light chain of clathrin as internal control because of its stable expression across tissues and treaments. Fold changes in gene expression were calculated as ratios of normalized values for each group over those of the saline group.

**Table 1:** Primer sequences.

Gene	Forward primer	Reverse primer
**Atf1**	GAT GCT CAA GGA AAC GGA	CAC ACA ACA CAC ACA GAA
**Atf2**	TCA TAA AGA TTG CCC TGT AAC	GAA CTG ACT CCA TTG GAC
**Atf3**	TGG AGT CAG TCA CCA TCA A	CAT TCA CAC TCT CCA GTT
**Atf4**	TCG GCC CAA ACC TTA TGA	TAG CTC CTT ACA CTC GC
**Atf5**	AGA AGA GAG ACC AGA ATA AG	CAT ACT GGA TCT CCC GT
**Atf6**	AAG TGA AGA ACC ATT ACT TTA TAT C	TTT CTG CTG GCT ATT TGT
**BiP/GRP-78**	TAC TCG AAT TCC AAA GAT TCA G	TCA AGC AGA ACC AGG TC
**P58^IPK^**	GAG CCC GAC AAT GTA AA	AAT AAT CCC GCT TCT GTG
**CHOP/Gadd153**	GGA AGT GCA TCT TCA TAC ACC ACC	TGA CTG GAA TCT GGA GAG CGA GGG
**Gadd34**	TGA ATG TTG AGA GAA GAA CC	TTG TTT AGA AGT CGC TCT G
**Clathrin**	AAG TAT CCG TAA GTG GAG	GGG GTT AAA GTC ACA CAG

### Western Blot

Cytoplasmic and nuclear fractions from striatal tissues were prepared using the NE-PER nuclear and cytoplasmic Extraction kit (Thermo scientific Pierce, Rockford, IL, USA). Protein concentration of cell lysates was quantified with the BCA protein assay kit (Thermo scientific Pierce, Rockford, IL, USA). For each protein studied, we have performed western blot analysis using six samples per group, and the experiment was replicated twice. Striatal protein lysates were separated by SDS-PAGE and electrophoretically transferred on PVDF membranes. Subsequently, the membranes were incubated overnight at 4°C with the following antibodies: HSP40, HSP70, Bad, Bax, Bcl-2, cytochrome c (1∶1000; Cell Signaling Technology Inc., Danvers, MA, USA), caspase-12 (1∶1000; Biovision, Mountain View, CA, USA), ATF3, ATF4 and CHOP (1∶200; Santa Cruz Biotechnology Inc., Santa Cruz, CA, USA). After incubation with the antibodies, the blots were washed with tris-buffered saline with 0.1% Tween-20. Afterwards, the membranes were incubated with horseradish peroxidase (HRP)-conjugated anti-rabbit/mouse secondary antibody (1∶1500; Cell Signaling Technology Inc., Danvers, MA, USA) for 1 hr at room temperature. To confirm equal protein loading, the blots were re-probed with α-Tubulin antibody (1∶4000, 2-hr at room temperature; Sigma-Aldrich, St. Louis, MO, USA34). LumiGLO chemiluminescent reagents (Cell Signaling Technology Inc., Danvers, MA, USA) were used to detect protein expression. Signal intensity was measured densitometrically with Carestream Molecular Imaging software (Carestream Health, Rochester, NY, USA).

### Statistical analysis

Statistical analysis for the qPCR and western blot data was carried out by a one-way ANOVA followed by post-hoc Fisher's protected least square difference (PLSD) test using StatView version 5.0.1 (SAS Institute, Cary, NC, USA). P values less than 0.05 were considered significant.
